# Preventing Importation of Poliovirus in the Horn of Africa: The Success of the Cross-Border Health Initiative in Kenya and Somalia

**DOI:** 10.4269/ajtmh.19-0040

**Published:** 2019-10

**Authors:** Ahmed Arale, Mercy Lutukai, Somane Mohamed, Lydia Bologna, Katherine V. Stamidis

**Affiliations:** 1 CORE Group Polio Project/Kenya and Somalia, Nairobi, Kenya;; 2 CORE Group Polio Project, Washington, District of Columbia

## Abstract

In 2013, the outbreak of wild poliovirus (WPV) in the Horn of Africa (HOA) triggered an aggressive, coordinated national and regional response to interrupt continued transmission. Kenya, Somalia, Ethiopia, South Sudan, and other HOA countries share a range of complex factors that enabled the outbreak: porous and sparsely populated borders, insecurity due to armed conflicts, and weak health systems with persistently under-resourced health facilities resulting in low-quality care and low levels of immunization coverage in mobile populations. Consequently, the continued risk of WPV importation demanded cross-border and intersectoral collaboration. Assessing and addressing persistent communication gaps at the subnational levels were necessary to gain traction for improved immunization coverage and surveillance activities. This article describes a systematic approach to institutionalizing processes of dialogue and facilitation that can provide for a sustainable and effective joint cross-border health platform between Kenya and Somalia. It examines an operational model called the Cross-Border Health Initiative (CBHI) to support joint intercountry collaboration and coordination efforts. To evaluate progress of the CBHI, the authors used data from population coverage surveys for routine immunization and supplemental immunization activities (for polio), from acute flaccid paralysis (AFP) surveillance, and from plans developed by border districts and border health facilities. The project-trained community health volunteers have been a critical link between the hard-to-reach communities and the health facilities as well as an excellent resource to support understaffed health facilities. The authors conclude that the CBHI has been effective in bolstering immunization coverage, disease surveillance, and rapid outbreak response in border areas. The CBHI has the potential to address other public health threats that transcend borders.

## INTRODUCTION

For more than three decades, humankind has pursued the possibility of a “polio-free world.”^1^ However, outbreaks following wild poliovirus (WPV) importations into previously polio-free countries remain an ongoing risk; this risk disappears only when polio is eradicated.^2^ Even after gaining polio-free certification, countries have struggled to remain polio-free. In 2010, the first WPV importation into the European region since the region was declared polio-free in 2002 resulted in 476 confirmed cases: 458 in Tajikistan, 14 in Russia, three in Turkmenistan, and one in Kazakhstan. In Africa and Asia, 11 new importations into six countries were observed in 2010; 30 WPV importations during 2008–2009 resulted in 215 WPV cases in 15 African countries during 2009–2013. Across six African and Asian countries, 11 new importations were recorded in 2009–2010.^3^

Kenya and Somalia have not been spared from the impact of WPV importation.^4^ In Somalia, the first importation occurred during 2005–2007, resulting in more than 200 cases of paralytic polio.^5^ Likewise, in Kenya, two cases in Garissa County were reported in 2006, 19 cases in Turkana County in 2009, and one case in Rongo district in 2011. Somalia experienced a polio-free period from 2007 to 2013.^5^ This period of calm was upset when an outbreak of WPV type 1 (WPV1) rattled the Horn of Africa (HOA).^6^ In May 2013, the Somalia Ministry of Health (MOH) and the WHO reported a confirmed WPV1 case in a child from Mogadishu (Banadir region). Subsequently, in May 2013, eight additional WPV1 cases were confirmed in Somalia: seven in the Banadir region and one in the Bay region.^7^ Just 3 weeks after the initial polio cases were detected in May 2013 in Somalia, Kenya reported its first case across the border in the Dadaab refugee camp. As a result, five people were paralyzed by polio, including young adults, in the northeastern part of Kenya.^8^ Polio quickly spread from Somalia to its neighboring countries of Ethiopia and Kenya. By April 2013, the case count stood at 223: 199 cases in Somalia, 14 in Kenya, and 10 in Ethiopia. All the Somalia polio cases belonged to cluster N5A,† which was known to have been circulating in northern Nigeria since 2011.

The 2013 HOA outbreak vividly confirmed that “All countries will continue to have some level of risk for WPV outbreaks as long as endemic circulation continues in Afghanistan, Nigeria, and Pakistan.”^9^ At the same time, the Global Polio Eradication Initiative entered a new phase, with a significant reduction in cases in endemic countries and a heightened recognition of the risk for the international spread of the virus.^10^ To combat the threat of an international outbreak, the WHO declared polio a public health emergency of international concern in May 2014 and issued recommendations requiring proof of polio vaccination for travel to and from countries experiencing polio cases.^10^ Frequent cross-border movement of the high-risk mobile populations between Kenya and Somalia and the low level of population immunity in the region continue to be major contributing factors to the spread of poliovirus and the risk of ongoing transmission.^11^

In 2014, Kenya’s MOH requested the United States Agency for International Development (USAID)-funded CORE Group Polio Project (CGPP) Kenya and Somalia HOA Secretariat based in Nairobi, Kenya, to initiate polio eradication activities in five counties along the Kenya–Somalia border deemed at high risk for poliovirus importation. It was clear that effective immunization activities across borders and migration pathways were essential to improve immunization rates. Under the leadership of the respective MOHs of Kenya and Somalia and in collaboration with the WHO, the CGPP began holding cross-border meetings in October 2014—a significant and instrumental move that would shape a systematic, unified, and well-coordinated response in the form of the Cross-Border Health Initiative (CBHI).^12^

Objectives arising from the 2015 cross-border meetings targeted improving collaboration between the health and administrative authorities of border regions through enhancing acute flaccid paralysis (AFP) surveillance sensitivity, increasing coverage of supplemental immunization activities (SIAs), and improving coverage and access to quality routine immunization services in the HOA border regions. Before the formation of the CBHI, cross-border committee meetings were first initiated by the WHO in collaboration with the Intergovernmental Authority on Development (IGAD) under the “Health for Peace Initiative” in 1996. However, these cross-border committees were formed in only a few selected sites and the meetings were ad hoc, resulting in limited capacity for implementation, monitoring, accountability, resource allocation, and sustainability of cross-border polio eradication activities. To address these gaps yet keeping and reaffirming the same set of valuable original objectives, the CGPP Kenya and Somalia HOA Secretariat over a 1-year period transformed the ad hoc cross-border meetings into a full CBHI in October 2015. The secretariat subsequently established more CBHI committees in Kenya and Somalia. The work of the committees funded by the CGPP set the course for the eventual full implementation of the CBHI.

The overarching goal of the CBHI is to reach every child with polio vaccine.^13^ Cross-border coordination bridges the disease surveillance gaps by forming partnerships among institutions, agencies, and communities in cross-border areas. Specifically, the CBHI works to ensure the vaccination of all cross-border populations, to support the detection of cases of AFP, to conduct joint case investigations of transborder AFP and WPV cases, and to synchronize all polio SIAs.

### The Cross-Border Health Initiative.

In October 2015, the CGPP established a total of seven CBHI committees in five polio high-risk counties‡ in Kenya and two regions of Somalia. Results of a risk assessment identified Turkana, Garissa, Wajir, Marsabit, and Mandera counties in Kenya for inclusion in the CBHI; all share a border with either South Sudan, Somalia, Ethiopia, or Uganda’s northern region. Based on the risk assessment, the CGPP established more committees in Kenya’s 14 subcounties bordering Somalia and Ethiopia§; in Somalia, the CGPP selected six districts from the Gedo and Lower Juba regions‖ for committee work. The MOH officials from the respective governments provided the leadership for the establishment of the CBHI and the formation of the committees, with funding from the CGPP.

In Kenya, the government-led committees consisted of representatives from the five counties and subcounties. At the county level, the representatives included the director for health, the disease surveillance coordinator, the Expanded Program on Immunization (EPI) coordinator, the health records and information officer, and the community health strategy focal person. At the subcounty level, representation included subcounty coordinators and disease surveillance or EPI officers. In addition, the CGPP implementing partner officers, WHO and United Nations Children’s Fund (UNICEF) county coordinators, and officers from border administration, immigration, and security completed the committee membership.

In Somalia, the committees consisted of regional and district medical officers and a regional EPI coordinator. Similar to the Kenya committees, representation included the CGPP implementing partner officers, WHO regional and district coordinators, UNICEF field staff, and officers from border administration, immigration, and security.

To reach high-risk mobile populations, the CGPP, in collaboration with the local authorities and CBHI, identified and profiled both formal and informal crossing points and communities at borders, transit hubs, and migratory routes. This exercise was beneficial; after developing detailed micro-planning to identify and document all border towns, villages, and settlements and their inhabitants, it was possible to estimate the number of children in the catchment areas. Moreover, the major transit points in the area provided pertinent information about population movement patterns, whereas joint mapping of border crossing points and border communities served to improve micro-planning for country-specific and joint country cross-border activities. The CGPP’s non-governmental organization (NGO) implementing partners and local health authorities mapped 11 border health facilities, 161 formal/informal border crossing points, and 372 villages along the border as shown in Table 1. The estimated population of children younger than 5 years is 557,036 and of children younger than 1 year is 120,068.

**Table 1:** Mapping border crossing points, border villages, and population sizes

Location of the CORE Group Polio Project in Kenya and Somalia	Number of sub-counties/districts	Number of border health facilities	Number of crossing points	Number of border villages	Estimated population of children < 1 year of age	Estimated population of children < 5 years of age	Bordering countries
Turkana	4	19	30	31	12,768	71,421	Ethiopia, Uganda, and South Sudan
Marsabit	2	18	18	22	5,345	30,747	Ethiopia
Garissa	3	9	9	37	8,823	47,168	Somalia
Wajir	3	16	26	41	15,460	67,050	Ethiopia and Somalia
Mandera	7	22	53	124	53,620	230,952	Ethiopia and Somalia
Lamu	1	6	7	16	310	1,101	Somalia
Lower Juba region	2	6	9	34	10,254	41,211	Kenya
Gedo region	4	14	14	67	13,488	67,386	Ethiopia and Kenya
Total	26	110	161	372	120,068	557,036	

To guide the CBHI committee members, the CGPP Secretariat in coordination with the MOH wrote a terms-of-reference document to structure regular meetings and outline roles for reporting and for the implementation of action plans. This document defined how to develop the agenda, meeting reports with outcomes, and implementation of activities arising from the meeting. Cross-Border Health Initiative committees followed activity work plan templates to ensure monitoring and accountability through joint supportive health facility visits. The project trained and provided the committees with selection criteria for recruiting community health volunteers (CHVs), whose primary focus was to ensure efficient and sustainable community-based surveillance, and with secondary responsibilities of supporting the committee and health facilities to conduct community mobilization for routine immunization and polio campaigns. In turn, the committees provided training materials for border health facility staff and border CHVs, including reporting forms based on the Integrated Disease Surveillance Response Framework to identify key diseases.

### Progress in cross-border coordination.

The government-led high-level, intercountry cross-border forums and CBHI committee meetings funded by the CGPP and other partners have served as critical communication and community engagement platforms to raise issues of the risk of polio importation in the HOA. The respective local health authorities have owned the CBHI and provided the leadership in its implementation. As a result, the CBHI has led to improved immunization coverage and increased AFP detection among high-risk mobile populations, limiting the importation of WPV across borders.

A key success of the committee’s work has been the recruitment of CHVs^14^ who serve as a link between the hard-to-reach nomadic communities and the health facilities. The committees, with funds from the CGPP, trained the CHVs to conduct social mobilization, perform community-based surveillance on vaccine-preventable diseases, report suspected AFP cases from the community, promote routine immunizations, and track immunization defaulters. Community health volunteers reported all suspected AFP cases from conflict-prone and insecure areas inaccessible to trained health workers. Table 2 shows the number of AFP suspected cases reported by the CHVs over the 3-year period—highlighting the role of the community as an excellent resource to support AFP surveillance for the polio eradication program. A total of 827 CHVs were trained in AFP surveillance during the 4-year period. Community health volunteers detected 59 of 179, or 33%, of all suspected AFP cases during that time in the project area.

**Table 2:** Number of CHVs trained and outputs at border subcounties/districts in Kenya and Somalia, 2015–2018

	Number of subcounties/districts with CHVs recruited				Number of CHVs trained on AFP surveillance				Number of AFP-suspected cases reported by CHVs				Total number of AFP cases reported by subcounties/districts			
Year	2015	2016	2017	2018	2015	2016	2017	2018	2015	2016	2017	2018	2015	2016	2017	2018
Kenya	5	15	18	20	0	83	329	519	3	10	15	44	52	44	48	151
Somalia	6	6	6	10	107	107	171	308	0	9	12	15	37	26	32	28
Total	11	21	24	29	107	190	500	827	3	19	27	59	89	70	80	179

CHV = community health volunteer.

The risk of persistent threat of WPV importation demands cross-border collaboration and coordinated interventions. In response, the CGPP, in collaboration with the respective country’s MOH and WHO, supported more than 70 local and international cross-border meetings in the HOA to highlight this risk (see Table 3). These meetings resulted in harmonizing collective efforts to strengthen surveillance, routine immunization, and SIAs and to prepare a region-wide response to any polio outbreak. The CBHI has improved information sharing between countries on polio eradication by identifying and addressing surveillance and immunity gaps in high-risk mobile populations, and by developing micro-plans along the borders to synchronize SIAs along the borders.

**Table 3:** Number of CBHI meetings conducted in Kenya and Somalia, 2015–2018

	Number of established CBHI committees				Number of in-country CBHI meetings conducted				Number of joint CBHI meetings conducted				Number of action plans developed			
Year	2015	2016	2017	2018	2015	2016	2017	2018	2015	2016	2017	2018	2015	2016	2017	2018
Kenya	4	5	5	14	0	11	23	46	2	3	3	3	2	5	3	22
Somalia	2	2	2	6	0	15	18	24	0	2	2	2	0	2	2	18
Total	6	7	7	20	0	26	41	70	2	5	5	5	2	7	5	40

CBHI = Cross-Border Health Initiative.

The CBHI has expanded access to polio vaccination for high-risk mobile populations in under-resourced areas. The strategic and systematic collaboration of the cross-border health committees has improved access to vaccination for children on the move at formal and informal border crossing points, enhanced tracking of nomadic pastoralist communities, and improved reporting of suspected AFP cases. Table 4 shows the increased number of children vaccinated during the SIAs in the border areas between 2016 and 2018. The CGPP CBHI mapped 328 crossing points and 1,105 cross-border villages. The initial maps were received from the Kenya National Drought Management Agency and the Food and Agriculture Organization of the United Nations. The updated maps were shared with the respective organization for validation and updating. AFP surveillance was intensified, with the number of suspected AFP cases increasing from three in 2015 to 59 in 2018 (44 in Kenya and 15 in Somalia).

**Table 4:** Outcomes of the Cross-Border Health Initiative in the last supplementary immunization activity in Kenya and Somalia

Year	Cross-border mapping and profiling		Supplemental immunization activities					AFP surveillance
	Number of mapped cross-border villages	Number of mapped border-crossing points	Number children < 5 years of age vaccinated at crossing points	Number of children 0–11 months of age receiving a dose of polio vaccine for the first time	Number of zero doses given among children 12–59 months of age	Number of children < 1 year of age who received a dose of polio vaccine	Number of children aged 12–59 months who received a polio immunization	Number of AFP cases reported by border subcounties/districts
2016	185	65	12,090	114	2,038	28	9,910	3
2017	443	129	38,913	714	8,098	495	30,085	11
2018	477	134	69,289	4,069	28,577	115,933	2,135,009	53
Total	1,105	328	120,292	4,897	38,713	116,456	2,175,004	67

* Zero dose: any child younger than 2 weeks of birth who has not received oral polio vaccine.

Major obstacles to polio eradication efforts in the HOA and specifically at the security-compromised border areas have been inadequate health infrastructure, limited funding, poor health workforce management, and low-quality skills of health workers. There are inadequate government services in the border areas as the presence of government and NGO workers is restricted because of security risks, especially kidnappings. Polio funding has been critical to supporting the expansion of other health services.^15^ Table 5 provides a tally of the number of health staff who were trained in polio eradication and the number of health staff who received supportive supervision. These are both well-established and proven capacity-building interventions for improving performance through micro-planning, monitoring and evaluation, advocacy and social mobilization, and program review. The CGPP support has crossed over to benefit additional health programs such as maternal and child health interventions, laboratory services, infection prevention and control, micro-planning, monitoring and evaluation, advocacy and social mobilization, and program review.

**Table 5:** Number of supportive field visits provided to border health facilities and number of health facility staff trained on AFP surveillance in Kenya and Somalia, 2015–2018

Country	Number of border health facilities supported	Number of supportive supervision visits conducted	Number of health staff trained
Kenya	98	1,098	467
Somalia	17	498	266
Total	115	1,596	733

The government EPI and surveillance officers carry out their activities through direct, personal contact on a regular basis to guide, support, and assist designated health-care facility staff to become more competent in their immunization and disease surveillance services. In coordination with the National Vaccine and Immunization Program and the Disease Surveillance and Response Unit of the respective government, the CGPP mentored the immunization and surveillance managers on a monthly and quarterly basis to conduct on-the-job training of the service providers, which had a positive impact on the immunization program indicators and on the provision of all services in general. In the border areas of the HOA, the CGPP has provided technical and logistic support for district/subcounty training on the Reach Every District strategy, local micro-planning, data quality self-audits, and supportive supervision of routine immunization at health facilities and outreach sites.

**Figure 1.**
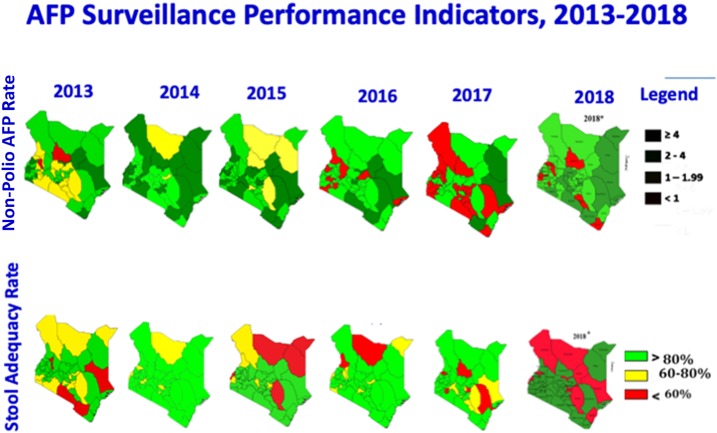
Kenya AFP surveillance performance indicators, 2013–2018. Source: Kenya Country Presentation to the 17th HOA Polio Technical Advisory Group on May 2–4, 2018.^16^

Cross-border coordination and scaled-up efforts of community-based surveillance activities to combat polio have contributed to positive AFP surveillance indicator trends; improvements are particularly significant in the hard-to-reach border areas (Figure 1). The geographic areas shown are Kenya’s counties. The northern border counties are supported by the CGPP. The first set of maps on the top row show the annualized nonpolio AFP rate per 100,000 children younger than 15 years: Kenya’s target is ≥ 4/100,000. The dark green in the legend shows more than or equal to 4, light green is 2–4, yellow is 1–1.99, whereas red is < 1. One can see a trend toward greater amounts of dark green moving from the left to right in Northern Kenya, which is supported by the CGPP.

The second row of maps shown in Figure 1 shows stool adequacy, defined as two adequate stools collected ≥ 24 hours apart and within ≤ 14 days after the onset of paralysis, the percentage of specimens arriving at a WHO-accredited laboratory within 3 days of being sent, and the adequacy of the volume of the stool specimen (8–10 g) that has been packed adequately and shipped in a cold box below 8°C. The target is ≥ 80%. The geographic areas shown are Kenya’s counties. The northern border counties supported by the CGPP have shown notable improvements in stool adequacy through 2017.

Multiple challenges have compromised the AFP surveillance efforts. A strike by health workers in Kenya in 2017 affected service delivery, particularly in the border counties of Turkana and Marsabit. The protracted conflict in South Sudan created an influx of refugees across the border in Kenya, further complicating surveillance efforts. The maintenance of biological specimens (in this case, stool samples collected from children with AFP) is referred to as the reverse cold chain. Maintenance of the reverse cold chain remains a significant barrier. Although stool sample collection rates are high, transportation of quality samples is still a problem due to poor infrastructure and impassable roads. Other operational challenges faced by the project were the high cost of programming due to the difficult terrain (poor roads and insecurity) and the occasional border closures hampering the cross-border work. However, some of these challenges were overcome by working closely with community leaders, building the capacity of the community through the CHVs to conduct the cross-border work, integrating the polio interventions with other cross-border activities undertaken by NGOs, and leveraging their presence and expertise. These partnerships created synergy and reduced the cost of programming. The CGPP closely coordinated with the IGAD, the regional intergovernmental body, and the respective immigration departments to address challenges with border access.

## DISCUSSION

The CGPP has demonstrated the value of an institutionalized approach that is collaborative and coordinated. This important strategy is adaptable to address other communicable diseases that transcend borders.^17^ The CGPP in the HOA has focused on improving access to vaccination services in high-risk mobile populations that straddle common borders. The CBHI in Kenya and Somalia has contributed to improved population immunity among cross-border communities, and it has created and maintained an active AFP surveillance system in these vulnerable regions that includes a community-based surveillance component. The CBHI has supported the collaboration of countries and border districts to develop early warning and response systems as well as the harmonization of cross-border vaccination and surveillance activities.^3^

There are several contributing factors that account for the early successes of the CBHI in Kenya and Somalia:

1. The communities that straddle the border of Kenya and Somalia are homogenous, sharing a common language and culture that allow for easier collaboration for teams on both sides of the border.
2. The contribution of communities through participation in the cross-border health committees and the community-based surveillance by CHVs has magnified the CGPP’s credibility and ownership among border communities.
3. The cross-border forums have heightened sensitization and advocacy for cross-border issues, leading to high-level political commitment and support from the respective government health authorities and renewed interest and support from the United Nations partners.
4. Frequent cross-border committee meetings (monthly, quarterly, and annually) have improved opportunities for communication, stronger partnerships, joint regional advocacy, effective management of challenges, and the facilitation and implementation of collective outbreak response activities.
5. Cross-border meetings have proven to be crucial at the local level, providing a forum to exchange information and carry out synchronized action for health service activities, including disease-control and eradication activities.
6. The CBHI has become vital for closely monitoring vaccination status to reduce the risk of WPV importation by those crossing the border. Effective communication among the cross-border committees and border authorities, coordinated immunization activities, and sharing of AFP surveillance data have led to an improved, in-depth understanding of the dynamics of cross-border threats of WPV importations.
7. The diverse committee representation of the stakeholders has ensured wide sharing of information across the borders.^18^
8. Implementation of the CBHI in Kenya and Somalia has strengthened information flow through monitoring and evaluation of joint action plans.
9. The border health facilities have conducted mapping and profiling of cross-border populations for routine immunization and SIA micro-planning in hard-to-reach border villages and in high-risk populations that include those residing in “no-man’s land” villages, immigrants and pastoralists, and nomadic populations.^19^
10. The capacity built through these committees, although specific to polio eradication, can serve other health initiatives, including the identification of emerging infectious disease threats with epidemic or even pandemic potential and other diseases that continue to challenge fragile health systems. This knowledge has been captured in the CBHI Operational Guide (contained in the online supplement). The guide promotes internal and cross-border partnerships between border health operational units to identify and address the health issues of border populations, identify and document transit routes and hubs, and monitor population movements between borders which can affect cross-border transmission of communicable diseases, including polio.^20^

As a testament to the power of collaboration, the CBHI’s domain has expanded to integrate the control of other infectious diseases.^1^ In 2016, the health committees in Turkana and Garissa counties added a country coordinator for tuberculosis to promote discussions with Uganda and Somalia on tuberculosis (TB) management. In Mandera County, the health committee worked together on a cholera outbreak in 2016. In addition, the Kenyan counties that border Somalia have merged their county development integrated plans with cross-border county action plans and widened the role of their CBHI committees to address other diseases. The CBHI is now an integral part of the operational health plans of border health units and the annual work plans of the participating county departments of health, thus ensuring sustainability of the CBHI after the end of the project. A manual that is used to guide the work of the CBHI is available as an online supplement to this article.

The CBHI has improved access to health services, particularly for the immunization of nomadic pastoralists. In partnership with other government departments and NGOs that provide nomadic communities with livestock, education, drought management, and internal security, the CBHI has successfully tracked the movement of the nomadic populations to vaccinate missed children—something that was unheard of before the CBHI. Even with the CBHI in the HOA, the long border between Kenya and Somalia remains porous and dangerous. The limited health infrastructure on the border there poses a significant challenge to effective cross-border collaboration. The CGPP will continue to build on the lessons of the CBHI to combat importation of WPV across the borders and will continue its search for new and innovative solutions.

In an acknowledgment that “Polio will not end everywhere until everywhere ends it,”^19^ the CBHI is an important step for “planning for the end of all polio.”^21^ The CBHI demonstrates the coherent impact of CBHI committees to engage across borders and across sectors (such as veterinary services and surveillance for zoonotic diseases) to capitalize on already existing synergies. The work of the committees as part of the CBHI could be adapted for other health interventions that require strong partnerships to achieve similar goals.

## CONCLUSION

Poliovirus circulation is most persistent in high-risk areas deprived of essential health services.^22^ The CBHI has been critical in reaching underserved, high-risk mobile populations with oral polio immunizations and other health services. Building on the success of the CBHI in Kenya and Somalia, this article highlights the value of the systemic process of institutionalizing the intervention so that it will continue beyond the eradication of polio. Integration of the CBHI committees into the country-driven County Integrated Development Plans has given particular attention to the often marginalized, security-compromised, and neglected border areas, raising coverage and potentially reducing inequities in immunization services. This integration has enhanced accountability and commitment through resource allocation and sustainability of the CBHI, leading to a gradual improvement in AFP surveillance and in routine immunization uptake in border areas. The CBHI has provided an in-depth understanding of the threat of disease importation, and it has built a cross-border framework for tackling cross-border challenges.

It is essential, however, to acknowledge the everyday challenges faced by HOA countries. The frequent movement of mobile populations in geographic areas that are unsafe for immunization workers makes the achievement of high levels of immunization coverage difficult and increases the threat of re-importation of the WPV and other communicable diseases,^23^ hence the need for political commitment under the premise of the cross-border governance framework of the IGAD that could institutionalize the CBHI and standardize regional surveillance reporting tools.^20^ Effective, robust cross-border coordination of polio eradication activities requires a high level of political commitment, solid coordination, and mobilization of cross-border communities and their leaders.^24^ The potential of strong cross-border collaboration for health that the CGPP’s CBHI has initiated holds great promise not only for contributing to the final eradication of polio but also for addressing other public health priorities.

## Supplemental Appendix 1

Supplemental materials

## Footnotes

† † Wild poliovirus serotype genetic cluster.

‡ ‡ High risk: Susceptible counties to polio outbreaks that were determined through risk assessment. The risk assessment examined the counties’ routine immunization coverage, AFP surveillance performance, and coverage of the most recent SIA round.

§ § Kenyan subcounties selected are as follows: Wajir North, Wajir South, Wajir East, North Horr, Moyale, Loima, Turkana West, Kibish, Mandera East, Lafey, Mandera South, Dadaab, Fafi, and Hulugho.

‖ ‖ Somalia districts selected are as follows: Afmadow, Badhadhe, Dollow, Belet-Hawa, Elwak, and Bardere.
