# Supplementary material for: Preventing Importation of Poliovirus in the Horn of Africa: The Success of the Cross-Border Health Initiative in Kenya and Somalia
Source: Am J Trop Med Hyg. 2019 Oct;101(4 Suppl):100–6. doi: 10.4269/ajtmh.19-0040 (PMC6776092; doi:10.4269/ajtmh.19-0040)
Supplement: Supplementary file 1 [file tpmd190040.SD1.docx]

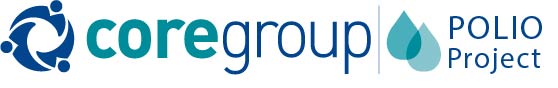


The Cross-Border Health Initiative

for Polio Eradication

OPERATION GUIDE


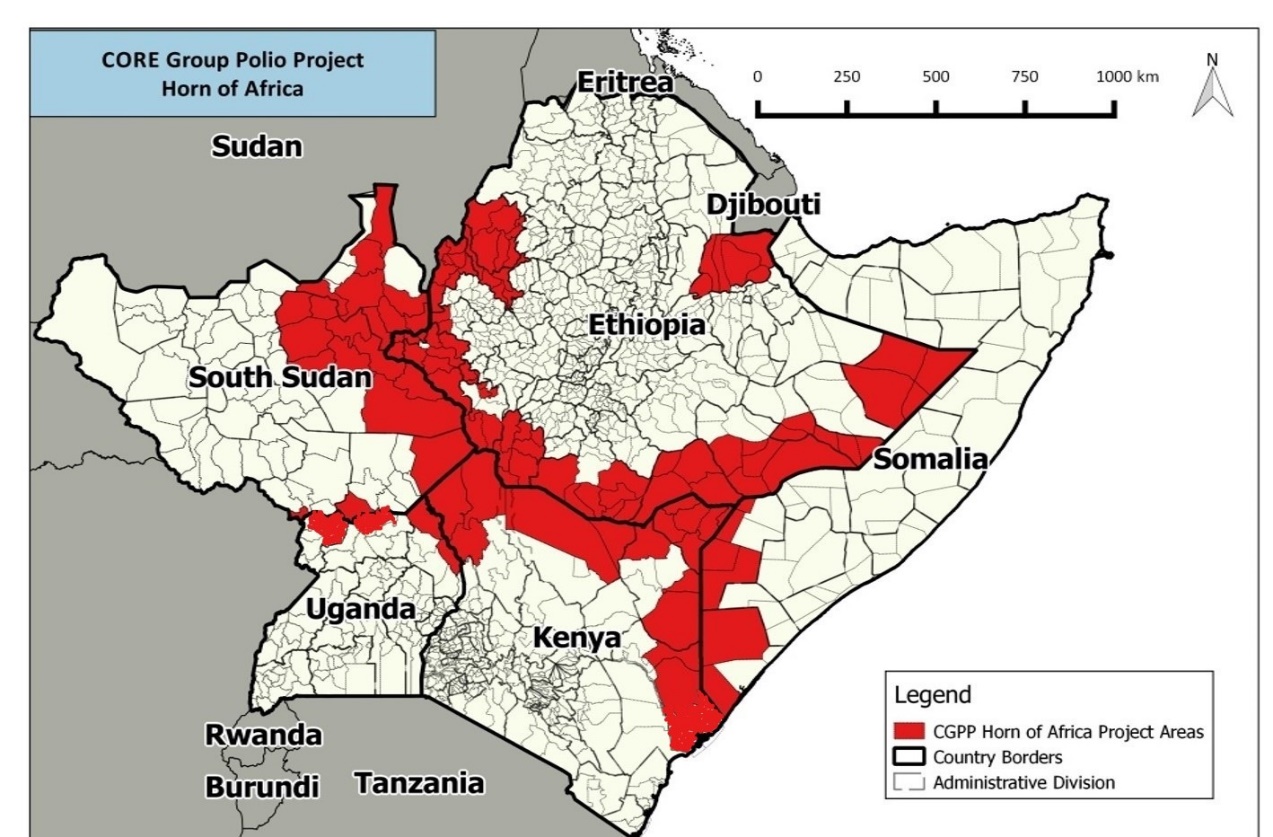


Developed by the CORE Group Polio Project

Horn of Africa Secretariat

Revised May 2019

**ACRONYMS**

AFP Acute Flaccid Paralysis

CBHC Cross-Border Health Committee

CBHI Cross-Border Health Initiative

CBO Community-Based Organization

CDC Centers for Disease Control and Prevention

CDSC County Disease Surveillance Coordinator

CGPP Core Group Polio Project

CHEW Community Health Extension Worker

CHO County Health Office

DHO District Health Office

EPI Expanded Program on Immunization

FBO Faith Based Organization

GPEI Global Polio Eradication Initiative

HOA Horn of Africa

HMIS Health Management Information System

HRMP High-Risk Mobile Population

IHR International Health Regulation

IGAD Intergovernmental Authority on Development

M&E Monitoring and Evaluation

MOH Ministry of Health

NGO Non-Governmental Organization

OPV Oral Polio Vaccine

RIGO Regional Intergovernmental Organization

SIA Supplementary Immunization Activities

TAG Technical Advisory Group

TB Tuberculosis

UNICEF United Nations Children’s Fund

USAID United States Agency for International Development

WHO World Health Organization

WPV World Polio Virus

Table of Contents

[BACKGROUND 1](#_Toc9446946)

[OVERVIEW OF THE CBHI OPERATION GUIDE 3](#_Toc9446947)

[UTILITY OF THE CBHI FOR POLIO ERADICATION 3](#_Toc9446948)

[GOAL 5](#_Toc9446949)

[OBJECTIVES 5](#_Toc9446950)

[COLLABORATING COUNTRIES 5](#_Toc9446951)

[INTERNATIONAL AND REGIONAL CROSS-BORDER HEALTH GOVERNANCE FRAMEWORK 5](#_Toc9446952)

[NATIONAL COMMITMENTS TO THE CBHI 5](#_Toc9446953)

[THE CROSS-BORDER HEALTH COMMITTEE 6](#_Toc9446954)

[MEMBERSHIP 6](#_Toc9446955)

[STRUCTURE 6](#_Toc9446956)

[MEETINGS 6](#_Toc9446957)

[COMMITTEE FUNCTIONS 7](#_Toc9446958)

[THE CROSS-BORDER HEALTH INITIATIVE PROCESS 8](#_Toc9446959)

[CBHI COMMITTEE MEETING 10](#_Toc9446960)

[CROSS-BORDER HEALTH SITUATION ASSESSMENT AND REPORT 11](#_Toc9446961)

[CROSS-BORDER DATA COLLECTION TOOLS 12](#_Toc9446962)

[JOINT ACTION PLANNING WORKSHOP 13](#_Toc9446963)

[IMPLEMENTATION, REVIEW, AND REPORTING OF JOINT ACTION PLAN 13](#_Toc9446964)

[Meetings 13](#_Toc9446965)

[MONITORING AND EVALUATION 14](#_Toc9446966)

[CBHI INDICATORS 14](#_Toc9446967)

[ANNEXES 15](#_Toc9446968)

[Annex 1: Cross-Border Community and Population Mapping and Profiling Tools 15](#_Toc9446969)

[Annex 2: Summary Analysis of Cross-Border Data Collection Tools 22](#_Toc9446970)

[Annex: 3 Cross-Border Workplan Template with Reporting Indicators 24](#_Toc9446971)

## BACKGROUND

Infectious disease outbreaks in the Horn of Africa (HOA) continue to be shaped by historical patterns of regional migration due to economic integration, socio-cultural practices of pastoralism and nomadism, and chronic issues of physical insecurity. Communities with large populations that move along and across formal and informal border points are highly vulnerable to the spread of infectious disease. These border communities have common risk factors: low population immunity, under-resourced health infrastructure with weak routine immunization systems, regional or local political instability, socioeconomic disadvantages, and a similar ecology. Hence, disease surveillance and rapid response to outbreaks is critical.

The 2013 outbreak of wild poliovirus (WPV) in the HOA countries triggered joint national and regional responses to interrupt the spread of transmission. Today, the flow of people and animals at the porous borders contributes to the threat of transmission of cross-border importations and epidemic outbreaks, particularly WPV. It also serves as an opportunity to reach at-risk children at or near the entry and exit border points with surveillance and immunization activities that are collaborative and coordinated.

The Global Polio Eradication Initiative (GPEI) in 2011 began cross-border interventions to improve cooperation, coordination, and collaboration between neighboring countries. At the center of this effort was the development of a Joint Strategic Action Plan for Polio Outbreak Preparedness and Response, with a specific focus on intensified AFP surveillance at the international, national and local borders. The HOA countries are considered as one epidemiological block. By using the existing Intergovernmental Authority on Development (IGAD) cross-border governance structures, an institutionalized cross-border health collaboration and coordination approach can be useful in not only interrupting the spread of the WPV, but it can be utilized for detecting communicable diseases that transcend borders.

Informed by recommendations from the 12th and 13th HOA Technical Advisor Group (TAG) meetings, the polio eradication partners developed cost-effective and efficient strategies to implement supplementary immunization activities (SIA), community-based AFP surveillance, and monitoring of cross-border activities. Effective communication between HOA countries and cross-border meetings have led to improved coordinated immunization activities, sharing of AFP surveillance data, and a keener understanding of cross-border population movement.

Between 2014 and 2018, the USAID-funded CORE Group Polio Project (CGPP), in collaboration with the respective MOHs and WHO, conducted more than 70 local and regional cross-border meetings in the HOA region to support the Joint Strategic Action Plan. To put the plan in action, discussions and planning sessions were held with border health administrators, surveillance and Expanded Program of Immunization (EPI) officers, border immigration and security personnel, and key GPEI stakeholders including UNICEF, WHO, CDC, and international and local Non-Governmental Organizations (NGOs). Extrapolating from these meetings and building upon the 11^th^ Technical Advisory Group (TAG) recommendation that called for standardising the process of cross-border activities, the HOA CGPP, with support from WHO, strategized to transform a series of ad-hoc cross-border meetings into a more sustainable, long-standing Cross-Border Health Initiative (CBHI) aimed to further direct, develop and document cross-border activities.

**THE CROSS-BORDER HEALTH INITIATIVE IS A CRUCIAL STRATEGY IN DISEASE SURVEILLANCE AND RAPID RESPONSE IN BORDER AREAS BY REQUIRING REPRESENTATION FROM KEY STAKEHOLDERS TO ENSURE SUSTAINABILITY, ACCOUNTABILITY, RESOURCES, ADEQUATE REPORTING, AND DOCUMENTATION**.

This CBHI Operation Guide is a product of these multiple efforts. While the CBHI emphasizes the critical need for cross-border collaboration, it also recognizes the equal importance of each border health authority to pay special focus and attention to its border communities, facilities and high-risk mobile populations (HRMPs) to mitigate the risk of cross-border importation of WPV.

The guide provides the following information: an overview of the CBHI; the process of organizing a CBHI meeting; the need to strengthen information sharing along and across borders; the implementation of the cross-border joint action plan; the development of a monitoring and reporting system of cross-border activities, and practical information on the aspects of effective partnerships that can be applied to cross-border initiatives in the Horn of Africa.


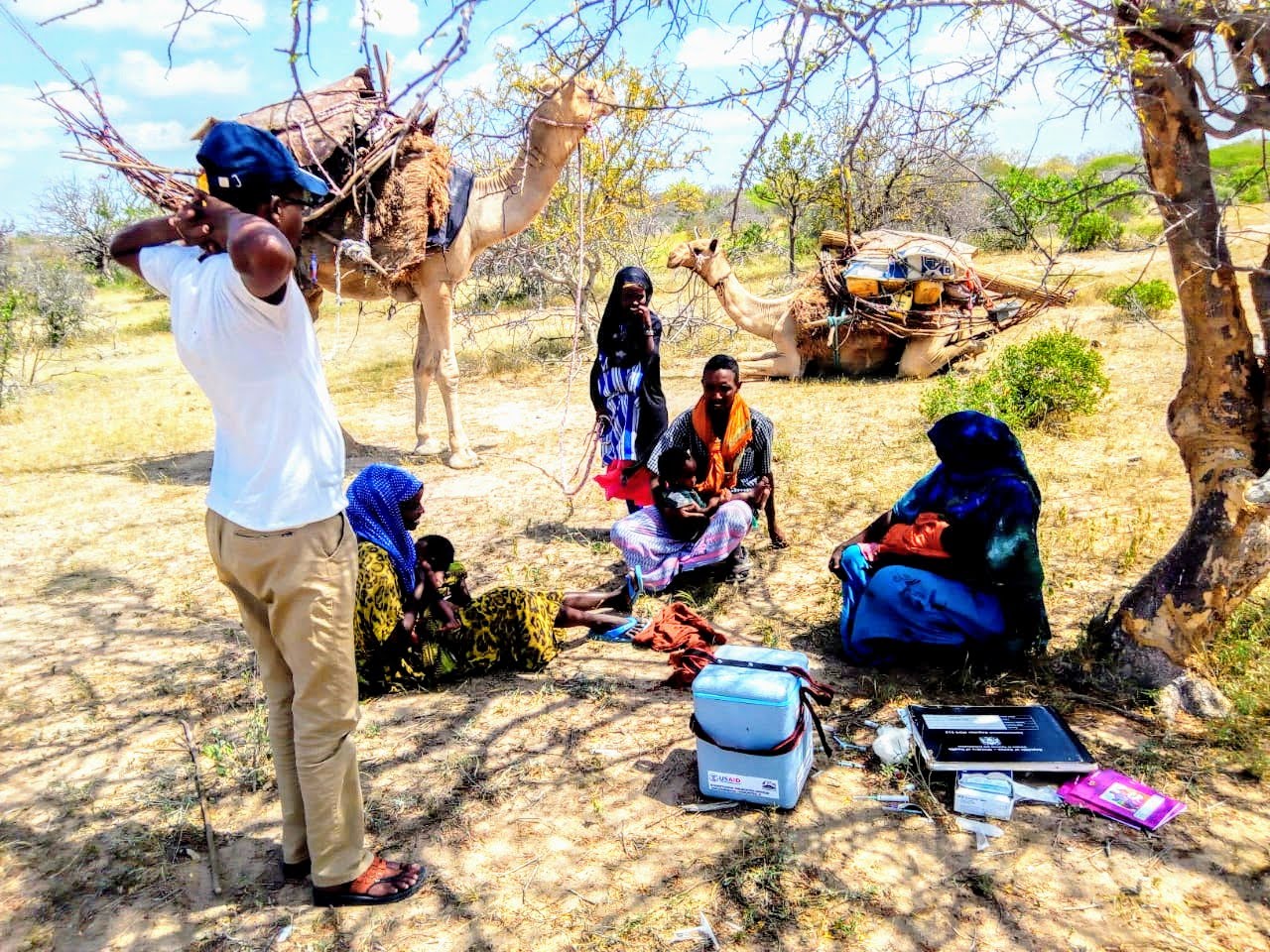


Figure 1: Targeted immunization and surveillance outreach for nomadic pastoralists along the Kenya-Somalia border.

Photo credit: Mohamud Amin-Program Officer-CGPP.

## OVERVIEW OF THE CBHI OPERATION GUIDE

This manual seeks to provide guidance on the planning and implementation process of cross-border health coordination activities. It also addresses the need for the reporting and documentation of these activities. The guide attempts to strengthen the strategic objectives for cross-border health undertakinigs into collective action by transforming cross-border health coordination meetings into a thorough and impactful process rather than a series of one-off or disconnected events. Its purpose is to internally promote cross-border partnerships between border health operational units to identify and address the health issues of the border populations, the transit routes and hubs, and the population movement between borders that affect cross-border transmission of communicable diseases including Polio. The Initiative has been expanded to integrate the control of other infectious diseases. In 2016, Turkana and Garissa counties in Kenya added a tuberculosis (TB) county coordinator to its CBHI committee to promote discussions with Uganda and Somalia. Meanwhile, Mandera County in Kenya leveraged the support of its CBHI committee to jointly respond to a Cholera outbreak with their counterparts in Somalia.

## UTILITY OF THE CBHI FOR POLIO ERADICATION

The purpose of the CBHI model is to support joint inter-country collaboration and coordination efforts in disease surveillance and response. Cross-border coordination bridges the disease surveillance gaps by forming partnerships among institutions, agencies, and communities in cross-border areas. Specifically, the CBHI works to ensure the vaccination of all cross-border populations; to support the detection of AFP cases; to conduct joint case investigations of trans-border AFP and WPV cases, and to synchronize all polio SIAs. This partnership between border health operational units (referred to as County/District Health Offices) works to identify and address health issues of border populations, to map transit routes and hubs, and to track the movement of populations to prevent or contain cross-border transmission of Polio and other communicable diseases. Collaborating governments are committed to the long-term priority of combating cross-border disease transmission disease through the investment of sufficient personnel and financial resources. The CBHI is designed to be part and parcel of the operational health plan of border health units and necessitates multiple steps of initiation, planning, execution, monitoring, and evaluation.

In 2014, Kenya’s MOH requested the USAID-funded CORE Group Polio Project (CGPP) Kenya and Somalia Secretariat based in Nairobi, Kenya to initiate polio eradication activities in five counties along the Kenya-Somalia border deemed high-risk for poliovirus importation. It was clear that effective immunization activities across borders and migration pathways were essential to improve immunization rates. Under the leadership of the respective MOHs of Kenya and Somalia, and in collaboration with WHO, the CGPP began holding cross-border meetings in October 2014 – a significant and instrumental move that would shape a systematic, unified and well-coordinated response and the eventual formation of the Cross-Border Health Initiative (CBHI).

The objectives from the 2015 cross-border meetings aimed to improve collaboration between the health and administrative authorities of border regions by enhancing AFP surveillance sensitivity, increasing coverage of Supplemental Immunization Activities (SIAs), and improving coverage and access to quality routine immunization services in the HOA border regions. Before the formation of the CBHI, cross-border committee meetings were first initiated by WHO in collaboration with the Intergovernmental Authority on Development (IGAD) under the “Health for Peace Initiative “in 1996. However, these cross-border committees were formed in only a few selected sites and the meetings were ad hoc, resulting in limited capacity for implementation, monitoring, accountability, resource allocation and sustainability of cross-border polio eradication activities. To address these gaps and building upon the original set of objectives, the CGPP Kenya and Somalia HOA Secretariat over a one-year period transformed the ad hoc cross-border meetings into a full CBHI in October 2015. The Secretariat subsequently established more CBHI Committees in Kenya and Somalia. The work of the Committees was funded by the CGPP, setting the course for the eventual full implementation of the CBHI.

The CBHI is a crucial strategy in disease surveillance and rapid response in border areas by requiring representation from key stakeholders to ensure sustainability, accountability, resources, adequate reporting, and documentation. The Initiative is powered by a network of the national governments of Kenya, Somalia, Ethiopia, South Sudan, Uganda, DRC, the local border administration, the border health facility in charges, local and international health partners that encompass NGOs (national and international) and community-based organizations, and key influencers such as local leaders (political, church and traditional) and businesses. The network is essential to ensure that CBHI plans are fully considered, focused, resilient, practical and cost-effective.  The network’s contribution also reinforces the importance of lessons learned and shared feedback across borders to inspire future planning and thoughtful decision making.


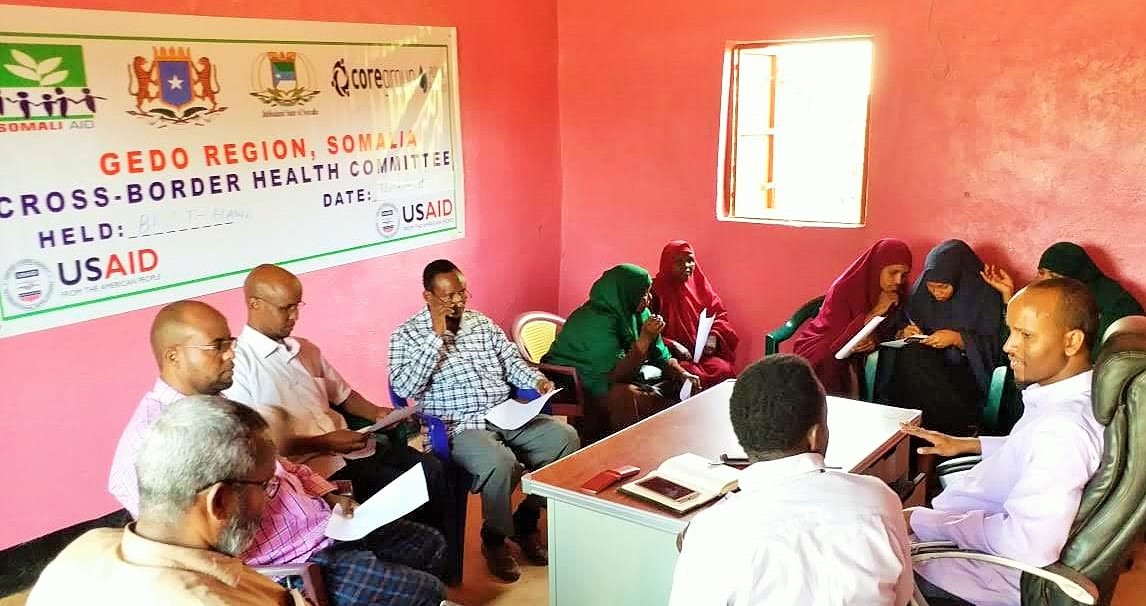


Figure 2: Gedo Region, Somalia: Cross-Border Health Committee meeting

GOAL

To contribute to the global eradication of polio by improving the population immunity in cross-border communities and cross-border populations with robust AFP surveillance and improved immunization coverage.

## OBJECTIVES

##

1. Improve cross-border collaboration between the health and administrative authorities of border regions
2. Establish a cross-border disease surveillance mechanism for polio and other diseases of public health importance
3. Harmonize cross-border vaccination and surveillance activities
4. Establish a mechanism to share disease surveillance data and joint disease investigation and response
5. Increase AFP surveillance sensitivity through the essential participation of cross-border communities and populations
6. Develop cross-border early warning and response systems for disease outbreaks

## COLLABORATING COUNTRIES

Ethiopia, Somalia, Kenya, South Sudan, and Uganda. CBHCs are currently operational in 5 Horn of Africa countries.

## INTERNATIONAL AND REGIONAL CROSS-BORDER HEALTH GOVERNANCE FRAMEWORK

In response to the increasing risks to population health posed by international travel and trade, and by emergence and re-emergence of infectious diseases, WHO developed the International Health Regulations (IHR) in 2005 to prevent, protect against, control and provide a public health response to the international spread of diseases. Regional Intergovernmental Organizations (RIGOs) notably IGAD, the eight-country economic block in the HOA, has developed a cross-border governance framework among its member countries to coordinate the prevention, control, and response to priority transboundary diseases in the HOA. Under the leadership of IGAD, the regional cross-border forums were held to develop a collective and coordinated response to the regional polio outbreak and put in place measures to standardize regional vaccination and surveillance tools.

## NATIONAL COMMITMENTS TO THE CBHI

The respective national Ministries of Health provide leadership and oversight of the CBHI, while the border Counties/District health offices manage the Initiative at the operational level. The operational level CBHI comply with relevant national policy and strategies on cross-border issues.

## THE CROSS-BORDER HEALTH COMMITTEE

The government-led Cross-Border Health Committee (CBHC) is the driving force of the CBHI. The CBHC represents the commitment of collaborating cross-border health offices to deal with common cross-border health issues. The CBHC is composed of key representatives from health offices, immigration, and the security sector. The number of members is typically equal to the collaborating border health offices, and its chairmanship is shared. On either side of the border, health officials should prioritize border health concerns and establish its committee to oversee issues internally.

### MEMBERSHIP

Committee members should represent various operational levels of the County Health Office/District and Sub-county/Sub-District Health Offfices (DHOs) of the collaborating countries:

1. CHO/DHO – 2 members
2. Sub-county/sub-DHO – 1 member
3. Stakeholder or partner – 1 member*
4. Immigration office – 1 member*
5. Security office – 1 member

*these members are invited to meet when agenda items are of concern, especially on disease outbreak response activities

At the county level, representatives included the Director for Health, the County Disease Surveillance Coordinator (CDSC), the Expanded Program on Immunization (EPI) Coordinator, the Health Records and Information Officer, and the Community Health Strategy focal person. At the sub-county level, representation include Sub-County Coordinators and Disease Surveillance or EPI Officers. Additionally, the CGPP implementing partner officers, WHO, and UNICEF county coordinators, and officers from border administration, immigration and security completed the Committee membership.

### STRUCTURE

*Co-chairpersons:*  Collaborating countries will designate two of its members to lead the committee for six months.

*Notetaker:* The committee will designate a notetaker from the collaborating countries for six months.

*Focal person*: The committee will designate a focal person from collaborating countries for six months to facilitate regular communication and information sharing.

### MEETINGS

The CBHC will meet monthly, quarterly, annually and minutes will be shared with regional counterparts. The CBHC will alternate chairmanship and venue between collaborating countries every six months.

1. Agendas, minutes and relevant documents for each meeting will be prepared jointly and shared with committee members electronically 14 days before the next meeting
2. Draft minutes will be distributed to committee members 14 days after the meeting. Minutes of the meeting will include committee discussions, decisions, and recommendations.
3. The CBHC meeting will include border health facilities in the semi-annual meeting to review and update for the country and joint action plans.

### COMMITTEE FUNCTIONS

- The cross-border health committee conducts a periodic situational analysis of the cross-border vulnerability of the spread of polio through the following steps:
  - Map the cross-border communities, migratory routes, cross-border entry/exit points, and transit hubs and routes for each of the cross-border facilities.
  - Document the risk factors of cross-border communities and populations of the spread of polio and other diseases of public health importance.
  - Assess the capacity of the border health facility to address border health issues.
  - Develop a situation analysis report.
- Organize a joint cross-border action planning workshop to develop joint and individual country annual work plans to address cross-border issues
- Update the micro plans for the delivery of routine immunization services at border health facilities
- Establish permanent or temporary transit vaccination posts.
- Update the micro plans for implementing supplementary immunization activities (SIAs) at border health facilities
- Establish weekly AFP zero reporting in border health facilities
- Develop an effective monitoring and reporting system for documenting and disseminating performance activities
- Support, document, and share the status of the implementation of the cross-border health action plan with county and national authorities
- Monitor and provide feedback on routine immunization, SIA, and AFP surveillance data from border health facilities
- Designate and share the contact information of a focal person for regular communication
- Share information on AFP surveillance with each other and with stakeholders
- Support and ensure border health facilities synchronize SIAs and surveillance activities.
- Develop a social mobilization and communication plan for increased demand and community participation among cross-border communities and populations in collaboration with respective border health facilities
- Support border health facilities to improve capacity, program coverage and quality of services for all cross-border communities and populations
- Supervise cross border health facilities with a focus on cross-border health issues
- Advocate to county administration and stakeholders for support and funding
- Hold a review meeting monthly by each country team member, sharing the minutes with the collaborating country
- Hold a review meeting quarterly by the cross-border committee, alternating the venue between countries

## THE CROSS-BORDER HEALTH INITIATIVE PROCESS

| **STEPS** | **BORDER County/DHOS** | **ROLE OF PARTNERS** | **MATERIALS NEEDED** | **NEXT STEP** |
| --- | --- | --- | --- | --- |
| Formal and Informal Consultation Between Border County/DHOs on CBHI | Review CBHI Operation Guide, Designate members for CBHC | Hold partners meeting by each County/DHO to discuss CBHI per review of Operation Guide | CBHI Operation Guide | Convene initiation meeting |
| Initiation Meeting | A County/DHO hosts the meeting, handles invitations | Partners organize and facilitate the meeting | CBHI Operation Guide, Cross-border Data Collection tools (paper and MS Excel), Workshop Delivery Guide | All parties agree to conduct cross-border situational assessment using the standard tools, agree to the next Joint Action Planning Workshop |
| Cross-border Health Situation Assessment | Each border County/DHO conducts a situation assessment using cross-border data collection tools, identifies problems, propose solutions | Partners support County/DHO in the assessment, analysis, and workplan development | Cross-border data collection tools | Each County/DHO prepares assessment report and workplan to present in the next Joint Action Planning Workshop |
| Joint Action Planning Workshop | A County/DHO hosts the meeting and handles invitations, presents assessment report and workplan | Partners organize and facilitate the meeting | Operation Guide, Completed Cross-border Data Collection tools (paper and MS Excel), Workshop Delivery Guide, Situation Assessment Reports, Workplan Template, Maps | Parties agree to implement the Joint Action Plan and next quarterly review meeting |
| Implementation of The Joint Action Plan | Each County/DHO implements its cross-border plan, participates in the joint cross-border action plan, documents and shares progress | Partners support DHOs in implementation, monitoring, documentation, and reporting | Workplan with the status of implementation of plan | Ongoing. Each county DHO convenes a monthly meeting to review the implementation and shares progress with counterpart border County/DHO |
| Quarterly Review Meeting | A County/DHO hosts the meeting and sends invitations, all County/DHOs share progress on implementation of the Joint Action Plan | Partners organize and facilitate the meeting | Joint Action Plans | Parties submit a report on the status of implementation and evidence to supporting partners and CBHI coordinating partners |
| Monitoring and Evaluation | Each border County/DHO establishes database and monitoring system, collects and documents data on indicators and report | Partners support County/DHOs in the design of the database, training on indicators, proper documentation of data and reports | Database and M&E System, Cross-border data collection tools | Ongoing |
| Annual Review and Planning | A County/DHO hosts the meeting and handles invitations. Each County/DHO prepares annual progress reports and plan for next year based on updated cross-border situation assessment | Partners take a supportive role as opposed to lead roles in previous meetings and workshop. County/DHO prepare annual reports, updating of situation assessment and development of the annual plan. | Annual status of implementation of CBHI plan and Joint Action Plan,  Updated Cross-border data collection tools,  Annual plan for the following year | The new year begins for implementation, periodic meetings, and reporting. |

## CBHI COMMITTEE MEETING

A formal invitation to join the CBHI is imperative as it demonstrates the Initiative’s important work and shows the involvement of other countries. The host county authority should prepare and send the letter once it is approved by current members. Details should include meeting dates, venues, objectives, and the names and designation of the invitees. It is prudent to also request that local border authorities inform their neighboring country and send a copy of the letter too. This reassures the neighboring country that they are welcome by their ‘neighbors.’ The letter should be sent two weeks prior to the CBHI meeting.

The one-day initiation meeting should be held at the hosting border CHO/DHO and meeting participants should be suggested by the CBHC. The CHO/DHO should also invite the focal person for the surveillance and immunization program and HMIS staff.

Suggested agenda

- Welcome and objectives
- Remarks for collaborating parties
- Presentation on status of polio eradication and other diseases or events
- Overview of CBHI
- Structure and functions of the CBHC
- Formation of CBHC
- Training on CBHI tools
- Overview of Joint Action Planning workshop
- Overview of action plan format for an accountability framework
- Next immediate steps
  - Cross-border health situation assessment and report
  - Joint Action Planning Workshop
- Close

The initiation meeting aims to create a CBHC which will oversee the overall process of cross-border collaboration. The participants will review the structure and functions of the cross-border committee and agree to any modifications. This involves reaching agreement with those in authority at the national, country local border health administrations, and key border local or International NGOs on special conditions or exceptions that include what the CBHI aims to achieve, the criteria and flexibilities involved in implementing the Initiative, resource mobilization or allocation, its parameters, scope, range, outputs, participants, budgets and timescales. Establishing and agreeing on the CBHI is an important process that should be done with great involvement of the country local border administrators on both sides for easier planning and future implementation of the Initiative.

In addition, the meeting is important in providing orientation on the overall process, tools and monitoring and evaluation of CBHI. Hence a training/orientation will be given to participants on cross-border tools so that they are able to complete, analyze and prepare a report on their cross-border health situation. The cross-border tools help document the border communities and populations at risk, identifying border entry and exit points and assessing the current capacity of border health facilities. (Refer to the annexes below for details on tools and training.) This will assist collaborating parties in identifying what needs to be done at the specific borders of interest through a proper examination of the current epidemiological situation and interventions of other health partners along the border of interest and decide on how best one can improve or enhance the interventions.

The meeting should conclude with the formation of the CBHC. At this point, participants should be trained on the use of cross-border situation assessment tools and provided hard and soft copies of these tools. Additionally, a timeline should be established for the completion of the assessment, the development of the report and the date of the joint action planning workshop.

## CROSS-BORDER HEALTH SITUATION ASSESSMENT AND REPORT

Following the initiation meeting, the collaborating parties should begin an assessment to analyze the past performance of border facilities on routine immunization, supplemental immunization coverage and disease surveillance based on the administrative data in the County/DHO. This assessment also collects data on catchment villages, entry and exit border points and the current capacity of each border facility. The border County/DHO staff responsible for cross-border collaboration will collect the required data for the situation analysis.

***The table on page 12 shows what, why and how data are collected as part of the cross-border situation analysis****.*

*
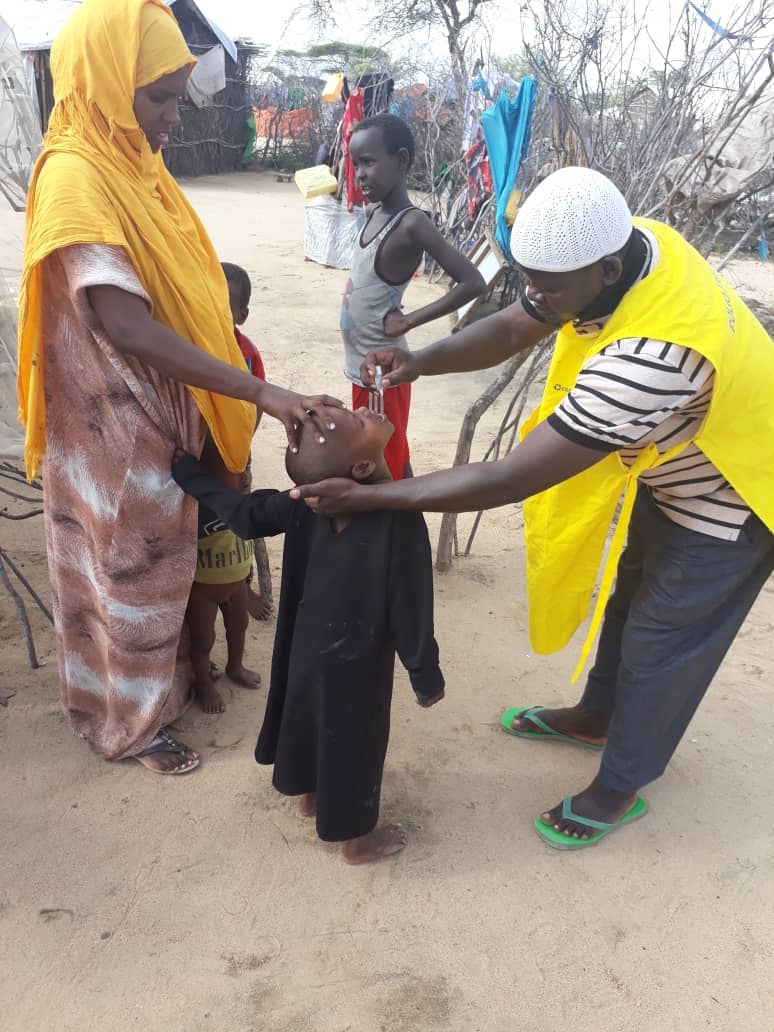
Figure 3: CORE Group Polio Project- trained volunteers in Somalia provide oral polio vaccine to children under five years old during a Supplemental Immunization Campaign.*

## CROSS-BORDER DATA COLLECTION TOOLS

| **Data** | **Purpose** | **Data collection and analysis tool** | **Source/method of collection** | **Who collects** |
| --- | --- | --- | --- | --- |
| Border Health Facility Capacity | To know the current capacity and gaps of the health facility to address cross-border health concerns | Yes | Self-Administered tools | Health Facility with assistance from trained staff from County/DHO at the health facility |
| Mapping and profiling of border communities and population | To understand the number, size, and distribution of villages and the relevant socio- economic, migration and health risk factors | Yes | Self-administered tools | Health Facility with assistance from trained staff from County/DHO at the health facility |
| Social map of catchment villages of health facility | To visualize the village in a spatial context and identify any problems | Yes, | Self-Administered | Health Facility with assistance from trained staff from County/DHO at the health facility |
| Mapping and profiling of cross-border points | To understand the number, size, and distribution of border crossing points and the current efforts and gaps in SIA | Yes | Self-administered tools | Health Facility with assistance from trained staff from County/DHO at the health facility |
| Mapping and profiling of transit hubs and routes | To identify village/towns/city in the district through which long distance migration takes place and to make a response plan for polio eradication | Yes | Self-administered tools | Committee members of County/DHO with assistance from the immigration office |
| Administrative Immunization data, SIA data, and Disease Surveillance data of border facilities | To identify problems pertaining to immunization coverage and surveillance | Yes | County/DHO | Committee members County/DHO |

Annex 1 contains a sample copy of tools and instructions on how to complete the form. Analysis of data includes summarizing the data and examining key data and indicators that define the current situations, problems, and next steps. A summary table summarizing data from various data collection tools are provided in Annex 2. A copy of all collected data, social maps, analysis, and workplan should be compiled into a single document. Each collaborating partner should use this data to identify the problem, develop solutions and create a draft work plan for review and discussion in a joint planning workshop. A workplan format is provided in Annex 3.

## JOINT ACTION PLANNING WORKSHOP

After each party has completed the situation assessment, a joint action planning workshop should be convened. The workshop should include all cross-border committee members as well as border health facility in-charges. The purpose of the joint action planning meeting is to apprise other parties of the cross-border issues and to individually and collaboratively address these issues. The workshop serves as an opportunity to assemble health facility staff to enhance local level understanding and increase collaboration.

Suggested workshop plan

- Welcome and objectives
- Remarks for collaborating parties
- Presentation on status of polio eradication
- Overview of CBHI
- Structure and functions of the CBHC
- Presentation of situation assessment and County/DHO cross-border workplan
- Joint Action Planning Work
  - Border County/DHOs share situation assessment findings and work plan; develop joint workplan
  - Border health facilities share data and consolidate jointly mapped crossing points into a single map; develop joint workplan
  - Documentation and exchange of contact details
- Documentation and reporting
- Close

## IMPLEMENTATION, REVIEW, AND REPORTING OF JOINT ACTION PLAN

Each party should implement its part of the joint action plan and is responsible for mobilizing its resources, with support from WHO, UNICEF, CGPP, and NGOs, to support the implementation of the workplan. Proper documentation on implementation should be filed as evidence and data on results is collected.

### Meetings

Each country’s or county/district team member of the CBHC should meet every month to review the workplan, document past activities, and prepare for activities to be held in the following months. Every quarter, the CBHC should meet jointly to share the progress of implementation of activities and provided in the given reporting format. The meeting will discuss success stories, challenges, and future plans. These meetings should be scheduled at least two weeks before SIAs to generate visibility and support for the campaigns. Review and planning should be conducted to assess annual progress and to develop the annual plan for next year.

## MONITORING AND EVALUATION

All collaborating countries should collect and document reports, training outputs, data and evidence against indicators listed in the workplan. At the end of the year, the cross-border data collection tools should be updated to reflect the changes. The supporting agencies should provide technical assistance to develop necessary M&E tools, database and in collection and management of the data needed for M&E of CBI. The supporting agency (i.e., WHO, CORE Group Polio Project) should provide technical assistance for verification of the reports and data.

## CBHI INDICATORS

- Number of border health facilities
- The total population covered by border health facilities
- % of border health facilities with a validated social map in the standard form
- % of border health facilities with Routine Immunization Micro Plan
- % of border health facilities with SIA Micro Plan
- Border DHO has mapped transit hub, and migration routes and a plan exist to address polio eradication issues.
- Number and % of border facilities with adequate cold chain
- Number and distribution of outreach routine immunization clinics
- Number and % of children vaccinated with routine Oral Polio Vaccine 0 (OPV 0) and OPV3 in border health facilities
- Number and % of children vaccinated with routine OPV 0 and OPV3 by outreach clinics in border health facilities
- % of outpatients of a border health facility that are from other countries.
- Number and % of children vaccinated in border health facilities during SIA
- Number of border entry and exit points identified formally and informally
- Reasons for crossing the border
- The estimated average number of commuters crossing the border daily
- Number and % of border crossing points covered for each SIA conducted
- Number and % border cross points synchronized for SIA
- Number of children vaccinated at border crossing points in each SIA conducted
- Number of children vaccinated at border points disaggregated by country of people
- Number and % of border health facilities submitting timely weekly zero surveillance reports
- Non-polio AFP rates from border health facility areas
- % adequate stool specimens collected from AFP cases reported by border health facility areas
- Border regions have a plan for cross-border health issues
- A functional CBHC exists
- % of work plan activities of CBHC implemented
- Number of CBHC organized by collaborating country border regions

## ANNEXES

### Annex 1: Cross-Border Community and Population Mapping and Profiling Tools

**1.1:** Mapping of catchment communities of border health facility

|  |  |  |  |  |  |  |  |  |  |  |  |  |  |  |  |  |  |  |  |  |  |
| --- | --- | --- | --- | --- | --- | --- | --- | --- | --- | --- | --- | --- | --- | --- | --- | --- | --- | --- | --- | --- | --- |
| **List all catchment villages/settlements/town of the border health facility. Include temporary population (nomad, pastoralist, IDP, refugee, slums, and squatters)** | | | | | | | | | | | | | | | | |  |  |  |  |  |
| *S/No* | *Name of Community on the border or within 10 km from the border* | *Type of settlement (Static or permanent, mobile, slum/squatters, or so on)* | *Estimated distance from the border (Km)* | *Name of Facility serving this community* | *Estimated distance from the nearest health facility (Km)* | *Estimated Population* | *Number of community health volunteers* | *Literacy status (below 33%, 34-66%, 67%+)* | *Religion (list in order of majority)* | *Occupation of the community (list in order of majority)* | *The community included in the RI micro plan? (Yes/No)* | *The community included in the SIA micro plan? (Yes/No)* | *Routine Immunization Coverage (below 60%, 60-79%, 80%+)* | *SIA coverage (below 80%, 80-89%, 90%+)* | *AFP cases ever reported in last 3 years? (Yes/No)* | *High risk? (Yes/No)* | *Hard to reach population? (Yes/No)* | *Does this community seek health service significantly from the other side of the border? (Yes/No)* | *Frequency of people crossing (Always, sometimes, Never)* | *Reasons for crossing the border (Trade, Grazing, Education, Health,)* | Name of CBO/NGO working in this community |
| **1** |  |  |  |  |  |  |  |  |  |  |  |  |  |  |  |  |  |  |  |  |  |
| **2** |  |  |  |  |  |  |  |  |  |  |  |  |  |  |  |  |  |  |  |  |  |
| **3** |  |  |  |  |  |  |  |  |  |  |  |  |  |  |  |  |  |  |  |  |  |
| **4** |  |  |  |  |  |  |  |  |  |  |  |  |  |  |  |  |  |  |  |  |  |
| **5** |  |  |  |  |  |  |  |  |  |  |  |  |  |  |  |  |  |  |  |  |  |

**1.2:** Mapping of Transit Hubs

| **List all hotels, lodges, communities, towns, and bus stations where people intending to cross the border stay temporarily (transit hubs)** | | | | | | | | | | | | |  |  |  |  |  |  |
| --- | --- | --- | --- | --- | --- | --- | --- | --- | --- | --- | --- | --- | --- | --- | --- | --- | --- | --- |
| *S/No* | *Name of building/area/bus station where the transient community are found in the transit hub* | *Type of transient community (Static or permanent, mobile, slum/squatters, or so on)* | *Name of the transient hub to which the transient community is accommodated* | *Estimated distance from the border (Km)* | *Name of the facility serving this community/transient hub* | *Estimated distance from the nearest health facility (Km)* | *Estimated Population of the transient hub* | *The estimated size of the population that these places can inhabit at a given time* | *Number of community health volunteers* | *The community included in the RI micro plan? (Yes/No)* | *The community included in the SIA micro plan? (Yes/No)* | *Routine Immunization Coverage of transit hub for the past 3 years (below 60%, 60-79%, 80%+)* | *SIA coverage of transit hub for the past 3 years (below 80%, 80-89%, 90%+)* | *AFP cases ever reported in last 3 years from bigger transit hub? (Yes/No)* | *High risk? (Yes/No)* | *Hard to reach population? (Yes/No)* | *Reasons for crossing the border (Trade, Grazing, Education, Health, etc.)* | Name of CBO/NGO working in this community |
| **1** |  |  |  |  |  |  |  |  |  |  |  |  |  |  |  |  |  |  |
| **2** |  |  |  |  |  |  |  |  |  |  |  |  |  |  |  |  |  |  |
| **3** |  |  |  |  |  |  |  |  |  |  |  |  |  |  |  |  |  |  |
| **4** |  |  |  |  |  |  |  |  |  |  |  |  |  |  |  |  |  |  |
| **5** |  |  |  |  |  |  |  |  |  |  |  |  |  |  |  |  |  |  |

**1.3:** Mapping Border-Crossing Points

| **Mapping of border crossing points** | | | | |  |  |  |  |  |  |
| --- | --- | --- | --- | --- | --- | --- | --- | --- | --- | --- |
|  |  |  |  |  |  |  |  |  |  |  |
| SN | Name of border crossing points | Name of the community along with the border crossing points | Name of a health facility that the crossing points belong to | Is crossing point formal or informal? | Name of the community along with crossing points on other side of the border | Name of a health facility that the crossing points belong to in other side of the border | The average number of people crossing the border in a day | Are animals crossing the border (Yes/No) | Are vehicles crossing the border? Yes/No | Is there an immigration check post on the border? |
|  |  |  |  |  |  |  |  |  |  |  |
|  |  |  |  |  |  |  |  |  |  |  |
|  |  |  |  |  |  |  |  |  |  |  |
|  |  |  |  |  |  |  |  |  |  |  |
|  |  |  |  |  |  |  |  |  |  |  |

**1.4:** Border Health Facility (HF) Capacity

| **Health Facility Capacity in serving the CBHI** | | | | | | | | | |  |  |  |  |  |  |  |  |  |  | | | | | | | |
| --- | --- | --- | --- | --- | --- | --- | --- | --- | --- | --- | --- | --- | --- | --- | --- | --- | --- | --- | --- | --- | --- | --- | --- | --- | --- | --- |
|  |  |  |  |  |  |  |  |  |  |  |  |  |  |  |  |  |  |  |  |  |  |  |  |  |  |  |
| S/No | Name of health facility | Catchment population | Number of catchment villages/settlements/towns | Number of sanctioned staff | Number of staff available | Is the staff responsible for immunization? (1=Yes, 0=No) | Is the staff responsible for surveillance? (1=Yes, 0=No) | Number of CHEWs | Type of health facility (Hospital, HC, Dispensary) | Ownership of health facility (Government, Private, FBO) | Means of transport within health facility areas | Population Literacy status (1= below 33%, 2=34-66%, 3=67%+) | Population Religion (list in order of majority) | Population Occupation of the community (list in order of majority) | Does the facility have adequate cold chain capacity? (1=Yes, 0=No) | Does the facility have a RI micro plan? (1=Yes, 0=No) | Does HF organize outreach clinics? (1=Yes, 0=No) | How many integrated/immunization outreach clinics the facility is currently operating? | Does the facility have SIA micro plan? (1=Yes, 0=No) | How many are SIA polio teams mobilized to cover the entire facility catchment area? | Number of community health volunteers in the facility catchment | Routine Immunization Coverage in past 3 years (1=below 60%, 2=60-79%, 3=80%+) | SIA coverage of in past 4 rounds (1=below 80%, 2=80-89%, 3=90%+) | Does the facility report weekly zero reports? (1=Yes, 0=No) | AFP cases ever reported in last 3 years? (1=Yes, 0=No) | Name of CBO/NGO working in this health facility catchment areas |
|  |  |  |  |  |  |  |  |  |  |  |  |  |  |  |  |  |  |  |  |  |  |  |  |  |  |  |
|  |  |  |  |  |  |  |  |  |  |  |  |  |  |  |  |  |  |  |  |  |  |  |  |  |  |  |
|  |  |  |  |  |  |  |  |  |  |  |  |  |  |  |  |  |  |  |  |  |  |  |  |  |  |  |

**1.5:** Mapping Crossing Points


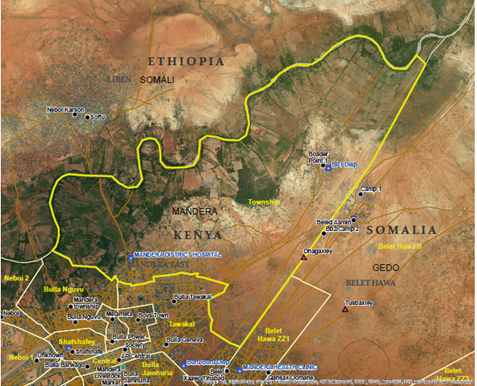

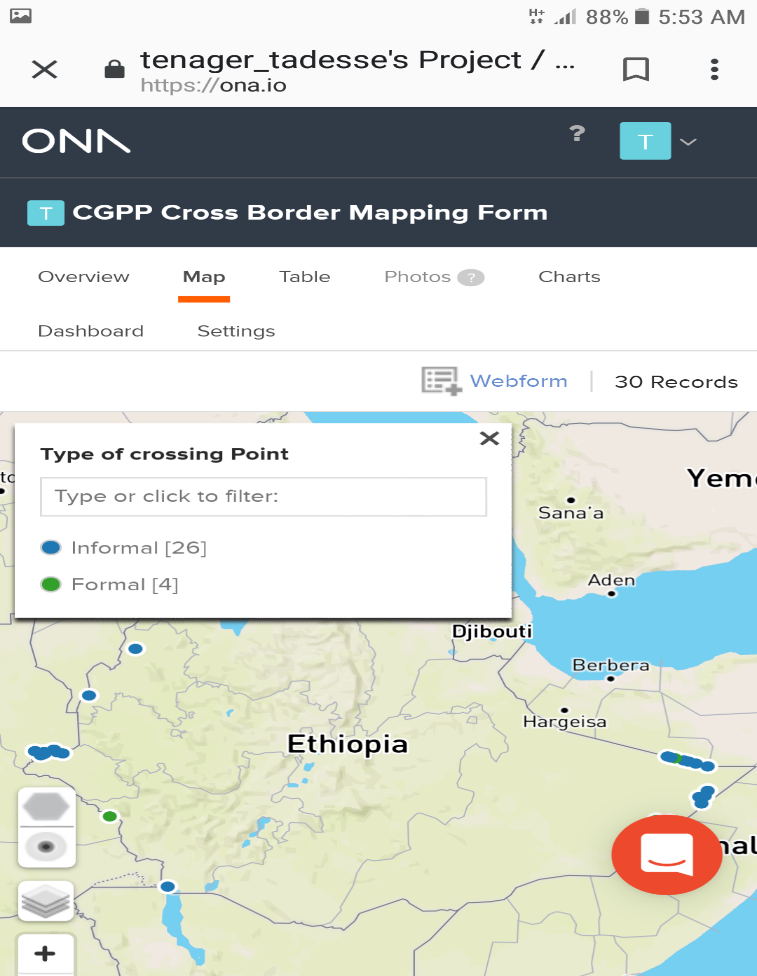

**1.6**: Social Map of Health Facility Catchment Area

**1.7**: Administrative Data of Border Health Facility on Polio Eradication

|  |  |  |  |  |  |  |  |  |  |  |  |  |  |  |
| --- | --- | --- | --- | --- | --- | --- | --- | --- | --- | --- | --- | --- | --- | --- |
| Name of border health facility | Population (Current year) | | | Routine Immunization Coverage (last year __________) | | | | Supplemental Immunization (last one ______) | | | | AFP Surveillance (Last year _____) | | |
|  | Population | Under 1-year Population | Under 5-year Population | OPV0 | OPV3 | Penta 3 | Measles | Target under 5 children | # of children vaccinated from house to house | # of children vaccinated outside the house | Number of vaccination teams employed | % weekly zero reporting completeness | AFP cases reported | Confirmed polio cases |
|  |  |  |  |  |  |  |  |  |  |  |  |  |  |  |
|  |  |  |  |  |  |  |  |  |  |  |  |  |  |  |
|  |  |  |  |  |  |  |  |  |  |  |  |  |  |  |
|  |  |  |  |  |  |  |  |  |  |  |  |  |  |  |
|  |  |  |  |  |  |  |  |  |  |  |  |  |  |  |
|  |  |  |  |  |  |  |  |  |  |  |  |  |  |  |
|  |  |  |  |  |  |  |  |  |  |  |  |  |  |  |
|  |  |  |  |  |  |  |  |  |  |  |  |  |  |  |
|  |  |  |  |  |  |  |  |  |  |  |  |  |  |  |
|  |  |  |  |  |  |  |  |  |  |  |  |  |  |  |

### Annex 2: Summary Analysis of Cross-Border Data Collection Tools

| **Summary of Situational Analysis** | | | | | | | | |  |  | | |  | |  | |  |  |  | |  | |  |  |
| --- | --- | --- | --- | --- | --- | --- | --- | --- | --- | --- | --- | --- | --- | --- | --- | --- | --- | --- | --- | --- | --- | --- | --- | --- |
| Table 1: Basic characteristics of border communities | | | | Table 2: Routine and SIA service coverage of border communities | | | | | | Table 3: Cross-border behaviors of border communities | | | | | | |  | Table 4: Health Facility staffing and types | | | | |  |  |
| Data | # | % |  | Data | | # | % | |  | Data | | | | # | | % |  | Data | | # | | % |  |  |
| Communities on the border or within 10 km from the border |  |  |  | Communities included in the RI micro plan | |  |  | |  | Communities that seek health service significantly from the other side of the border? | | | |  | |  |  | Number of sanctioned staff | |  | |  |  |  |
| Communities that are permanent |  |  |  | Communities included in the SIA micro plan | |  |  | |  | Communities that cross the border frequently | | | |  | |  |  | Number of staff available | |  | |  |  |  |
| Communities that are a mobile pastoralist |  |  |  | Communities with estimated routine immunization coverage below 60% | |  |  | |  | Communities that cross the border infrequently | | | |  | |  |  | Staff responsible for immunization | |  | |  |  |  |
| Communities that are a mobile nomad |  |  |  | Communities with estimated routine immunization coverage between 60% to 79% | |  |  | |  | Communities that never cross the border | | | |  | |  |  | Staff responsible for surveillance | |  | |  |  |  |
| Communities that are slums/squatters |  |  |  | Communities with estimated routine immunization coverage 80%+ | |  |  | |  | Communities that cross the border for trade | | | |  | |  |  | Number of CHEWs | |  | |  |  |  |
| Average of the Estimated distance of communities from the border (Km) |  |  |  | Communities with estimated SIA coverage below 80% | |  |  | |  | Communities that cross the border for pastures | | | |  | |  |  | Type of health facility (Hospital, HC, Dispensary) | |  | |  |  |  |
| Average of the Estimated distance of communities from the nearest health facility (Km) |  |  |  | Communities with estimated SIA coverage between 80% to 90% | |  |  | |  | Communities that cross the border for education | | | |  | |  |  | Ownership of health facility (Government, Private, FBO) | |  | |  |  |  |
| Estimated Total Population of communities |  |  |  | Communities with estimated SIA coverage 90%+ | |  |  | |  | Communities that cross the border for healthcare | | | |  | |  |  | Number of community health volunteers in the facility catchment | |  | |  |  |  |
| Communities with the Literacy rate below 33% |  |  |  | Community health volunteers | |  |  | |  | *Data source: mapping of communities* | | | |  | |  |  | Population Literacy status (1= below 33%, 2=34-66%, 3=67%+) | |  | |  |  |  |
| Communities with the Literacy rate of 33% to 66% |  |  |  | Communities that have reported AFP cases in the last 3 years | |  |  | |  |  | | | |  | |  |  | Population Occupation of the community (list in order of majority) | |  | |  |  |  |
| Communities with the Literacy rate of 67% |  |  |  | *Data source: mapping of communities* | | | | |  |  | | | |  | |  |  | *Data source: health facility capacity* | | | | |  |  |
| Communities that are High Risk for polio eradication |  |  |  |  | |  |  | |  |  | | | |  | |  |  |  | |  | |  |  |  |
| Communities that are Hard to Reach |  |  |  |  | |  |  | |  |  | | | |  | |  |  |  | |  | |  |  |  |
| *Data source: mapping of communities* | | |  |  | |  |  | |  |  | | | |  | |  |  |  | |  | |  |  |  |
| Table 5: Health Facility Routine Immunization and SIA capacity | | | | | | | | | | |  | Table 6: Number and characteristics of border crossing points | | | | | | | | | | | | |
| Data | | | | | Number | | | % (if applicable) | | |  | Data | | | | | | | | | | Number | | % |
| The health facility has adequate cold chain capacity | | | | |  | | |  | | |  | Number of border crossing points | | | | | | | | | |  | |  |
| Health Facility has a RI micro plan | | | | |  | | |  | | |  | Number of crossing points that are formal | | | | | | | | | |  | |  |
| Health facility organizes outreach clinics | | | | |  | | |  | | |  | The average number of people crossing the border in a day | | | | | | | | | |  | |  |
| Number of integrated/immunization outreach clinics the facility is currently operating | | | | |  | | |  | | |  | Number of crossing points with an immigration check post | | | | | | | | | |  | |  |
| Routine Immunization Coverage in past 3 years (1=below 60%, 2=60-79%, 3=80%+) | | | | |  | | |  | | |  | Number of crossing points with a permanent vaccination post at this crossing point | | | | | | | | | |  | |  |
| Health Facility has SIA micro plan | | | | |  | | |  | | |  | Number of crossing points with a static vaccination post from Kenya | | | | | | | | | |  | |  |
| Number of SIA polio teams mobilized to cover an entire facility catchment area | | | | |  | | |  | | |  | Number of crossing points with a static vaccination post from South Sudan | | | | | | | | | |  | |  |
| SIA coverage of in past 4 rounds (1=below 80%, 2=80-89%, 3=90%+) | | | | |  | | |  | | |  | Number of crossing points with a static vaccination post from Uganda | | | | | | | | | |  | |  |
| Health facility report weekly zero AFP surveillance report | | | | |  | | |  | | |  | Number of crossing points with a static vaccination post from Kenya and South Sudan | | | | | | | | | |  | |  |
| AFP cases ever reported in last 3 years | | | | |  | | |  | | |  | Number of crossing points with a static vaccination post from Kenya and Uganda | | | | | | | | | |  | |  |
| *Data source: health facility capacity* | | | | |  | | |  | | |  | Number of crossing points with a static vaccination post from South Sudan and Uganda | | | | | | | | | |  | |  |
|  | | | | |  | | |  | | |  |  | | | | | | | | | | | |  |

### Annex: 3 Cross-Border Workplan Template with Reporting Indicators

**Cross-border joint action plan implementation status reporting**

Instructions:

- Each collaborating county should complete the implementation status individually and share with others by email; copies should be shared with the relevant supporting partners.
- Choose one of the options provided in the implementation status. If the activity is completed, send a copy of supporting documents as listed in Means of Verifications. If not completed, provide an explanation under the comments section.

| **#** | **Issues** | **Planned Activities** | **Level of Implementation** | **Time frame** | **Responsible body/person** | **Monitoring Indicators** | **Means of verification** | **Implementation status (completed, on-going, not started, cancelled) as of__________ (Date)** | **Comments** |
| --- | --- | --- | --- | --- | --- | --- | --- | --- | --- |
| **A** | **Cross-border coordination & collaboration** | | | | | | |  |  |
| 1 | No designated County/DHO Cross-border Focal Person. | Designate cross-border focal person and share contact information  Develop a term of reference for Cross-border Focal Person | District/County |  | District/County Health Office | Name, position and contact address of designated cross-border focal person at CHOs shared between border counties.  Terms of Reference for Cross-border Focal Person | An official letter from CHO on the designation of cross-border focal person with approved Terms of Reference for Cross-border Focal Person |  |  |
| 2 | No cross-border committees in districts/counties | Establish district/county cross-border health committees to be chaired by DHO/CHO  Develop a term of reference for cross-border committees | District/County |  | District/County Health Office | List of names and positions nominated for cross-border committees from bordering counties.  Terms of reference for cross-border committee | An official letter from CHO designating staff members serving in cross-border committee and with approved Terms of Reference |  |  |
| 3 | Information not regularly shared among bordering districts/counties | Share information monthly by email and any other appropriate means using the agreed template | District/County | Every 2^nd^ week of the new month | District/County Health Office | Number of times the information was shared between cross-border focal persons of border counties through email or any other appropriate means | Copy of the information shared (emails, conference call notes) |  |  |
| 4 | No cross-border health committee meetings held for border districts/ counties | Hold quarterly cross-border health committee meetings  Develop a guideline for conducting cross-border quarterly meeting | District/County Health Office | Every 3rd week of the new quarter | District/County Health Office | Number of cross-border quarterly health committee meetings held following the guideline | Quarterly Meeting Reports as per the guideline  Pictures of meeting |  |  |
| 5 | No funding for cross-border activities. | Cost the Cross-border workplan  Identify the commitments from various sources – MOH, Partners | District/County |  | District/County | Cross-border workplan with costing  Amount and % of funds committed | Copy of costed cross-border workplans with funds commitments |  |  |
| 6 | Annual meetings not alternated between countries | Conduct annual cross-border meetings in alternative countries. Next meeting to be held in Uganda | Regional | Every August | MOH | Annual meeting held in alternate countries | Meeting reports  Pictures of meeting |  |  |
| 7 | Contact information not shared among key focal points at border health facilities | Share contacts of key focal points at all border health facilities and update as necessary | County/District |  | County/District health departments | Number of border health facilities that have shared contact information | List of focal points and their contact information available in a health facility |  |  |
| **B** | **Polio SIAs** | | | | | | |  |  |
| 8 | No synchronization of polio SIAs across the countries in the region leading to missed children | Implement synchronized polio SIAs between bordering countries in the region | Regional | Next rounds of polio SIAs | WHO | Number of countries in the region that have implemented synchronized polio SIAs | Technical reports |  |  |
| 9 | No mapping of border communities and crossing points for synchronization of SIAs | Each county conduct/update mapping of border communities and crossing points using standardized data collection tools.  Cross-border committee meet for planning, implementation, and sharing of reports for synchronized and collaborated SIA | District/County and border facilities | Next rounds of polio SIA | District/County Health Office and border facilities | A single border map showing border communities and crossing points between border county,  Data on border communities, crossing points, transit hubs and border health facility capacity in a given template, Micro plan to cover cross-border population, Jointly agreed plan on synchronized and collaborated action plan for SIA for cross-border population, number of children immunized by border communities, crossing points and transit hub | Border map with the location of border communities and other population of concern  Report on cross-border mapping  Pictures of data collection  Pictures of cross-border communities and population |  |  |
| 10 | Polio SIAs are not given prominence in some countries | Conduct special launching of SIA campaigns at the border areas by high government ranking officials | District/County and border facilities | Next rounds of polio SIA | District/County Health Office and border facilities | Number of cross-border points where joint SIA was launched, | Brief reports on launching ceremonies, Pictures of speakers and events. |  |  |
| 11 | Limited monitoring & supervision during SIAs at the bordering districts/counties | Strengthen supervision of SIAs at bordering districts/counties  Conduct Independent Monitoring in cross-border population | District/County and border communities | Next round of polio SIA | National, district and county authorities, and partners  Independent Monitoring Coordinator | Number cross-border communities supervised  IM conducted | Supervision reports  IM Reports  Pictures of supervision done  Pictures of IM done |  |  |
| 12 | Inadequate sharing of SIA and IM reports between Region s/States & districts/counties | Share SIA, and IM reports with districts/counties/national level | National /State/County/Cross-border committee | Next round of polio SIA | Regional/State authorities | On the third week of SIA round SIA and IM data is discussed in 1) Cross-border review meeting 2)County review meeting, 3)national review meeting | Meeting report of cross-border, county and national review meetings.  Pictures of meetings  Attendance of meetings |  |  |
| 13 | Everyday static polio vaccination posts not in place crossing points | Put an everyday static polio booth at major border crossing points | Border crossing points |  | County/District/border facility | Number of border crossing points with everyday static polio booth  Number of children immunized | Routine activity report |  |  |
| **C** | **Social Mobilization** | | | | | | |  |  |
| 14 | Social mobilization is not synchronized among the communities in border areas | Develop social mobilization plan for cross-border communities and points  Organize inter-border facility meeting to agree on to joint social mobilization plan | Border Facilities | Two weeks before next round of polio SIAs | County health office/border facility in-charge | Number of border facilities having a social mobilization plan  Number of border facilities sharing and agreeing to cross-border SIA social mobilization plan | A copy of the social mobilization plan of border facilities on both side of the border  Copy of joint social mobilization plan  Pictures of social mobilizations carried out  A report on the implementation of social mobilization activities |  |  |
| 15 | Messages are not translated into the local languages | Translate the standardized social mobilization messages into local languages | District/County | One month before next round of polio SIAs | District/County Health Office | A copy of messages translated into local languages | List of translated standardized social mobilization messages |  |  |
| **D** | **Surveillance** | | | | | | |  |  |
| 16 | Community-based disease surveillance is lacking in border areas | Launch community-based surveillance in border communities:  Selection of volunteers, training of volunteers, Information system, supervision system, reporting, and review system, motivation system | Border facility areas |  | District/County Health Office and border facilities | The proportion of districts and counties that have reoriented community resource persons on AFP surveillance | Monthly reports |  |  |
| 17 | AFP surveillance is weak in border areas | Ensure constant availability of surveillance reimbursement funds at UVR/DSRU Laboratory | District/County and border facility | October 2014 | District/County Health Office and border facilities | Number of weekly reported submitted by border facilities | Weekly reports filed at the facility  County health officials report log |  |  |
| 18 | The weak linkage between community AFP surveillance & other services | Strengthen linkage between community surveillance & other services | District/County and facility | Sept 2014 | District/County Health Office | Number of CSV supervised by health facility staff at least once in a quarter  Information on CBS (volunteers and cases reported) in the facility | Monthly reports  Health facility records on CBS |  |  |
| **E** | **Routine Immunization** | | | | | | |  |  |
| 19 | Cold chain equipment and other immunization resources gap in some border health facilities | Conduct an assessment of border health facilities for cold chain equipment and other resources for routine immunization | District/County and border facilities | October 2014 | District/County Health Office and border health facility | Number of border health facilities that had an inadequate cold chain for routine immunization service provision | Report on the assessment of the border facilities |  |  |
| 20 | Hard to reach/ unreached populations along the border not reached with routine immunization | Map all border communities  Review and update micro plan for routine immunization service delivery | District/ County and border facilities | October 2014 | District/County Health Office and border health facility | Map of all border communities  All communities are covered for routine immunization service provision  outreaches | Routine Immunization micro plan  Map of communities |  |  |
| **F** | **Monitoring &Evaluation** | | | | | | |  |  |
| 21 | Lack of reporting. | Facilitate the assessment and mapping of CBHI (tools gave) | District/county and border facilities | Oct 2014 | District/County Health Office and border facilities | Number of border facilities completed assessments | Baseline assessment report |  |  |
| 22 |  | Report quarterly on the implementation status of joint cross-border workplan  Include a copy of documents/supporting materials listed in Means of Verification | District/county | Oct 2014 | District/county health office | Number of quarterly reports  Completeness of supporting documents included | Reports and supporting documents |  |  |
| 23 |  | Document all reports, data, pictures as needed in means of verifications | District/county | Oct 2014 | District/County health office | Availability of storage and retrievability of supporting documents | Independent verification |  |  |
| 24 |  | Document SIA, RI, AFP surveillance data and survey (IM, Immunization) data on CBHI |  |  |  |  |  |  |  |
